# Polar Cooperative Navigation Algorithm for Multi-Unmanned Underwater Vehicles Considering Communication Delays

**DOI:** 10.3390/s18041044

**Published:** 2018-03-30

**Authors:** Zheping Yan, Lu Wang, Tongda Wang, Zewen Yang, Tao Chen, Jian Xu

**Affiliations:** College of Automation, Harbin Engineering University, Harbin 150001, China; yanzheping@hrbeu.edu.cn (Z.Yan); wangtongda@hrbeu.edu.cn (T.W.); yangzewen@hrbeu.edu.cn (Z.Yang); chentao_7777@163.com (T.C.); xujian_bsa@hrbeu.edu.cn (J.X.)

**Keywords:** underwater technology, marine navigation, adaptive filters, cooperative systems, polar region, Unmanned Underwater Vehicles (UUV)

## Abstract

To solve the navigation accuracy problems of multi-Unmanned Underwater Vehicles (multi-UUVs) in the polar region, a polar cooperative navigation algorithm for multi-UUVs considering communication delays is proposed in this paper. UUVs are important pieces of equipment in ocean engineering for marine development. For UUVs to complete missions, precise navigation is necessary. It is difficult for UUVs to establish true headings because of the rapid convergence of Earth meridians and the severe polar environment. Based on the polar grid navigation algorithm, UUV navigation in the polar region can be accomplished with the Strapdown Inertial Navigation System (SINS) in the grid frame. To save costs, a leader-follower type of system is introduced in this paper. The leader UUV helps the follower UUVs to achieve high navigation accuracy. Follower UUVs correct their own states based on the information sent by the leader UUV and the relative position measured by ultra-short baseline (USBL) acoustic positioning. The underwater acoustic communication delay is quantized by the model. In this paper, considering underwater acoustic communication delay, the conventional adaptive Kalman filter (AKF) is modified to adapt to polar cooperative navigation. The results demonstrate that the polar cooperative navigation algorithm for multi-UUVs that considers communication delays can effectively navigate the sailing of multi-UUVs in the polar region.

## 1. Introduction

Unmanned Underwater Vehicles (UUV) are important pieces of equipment used in ocean engineering for marine development and have made great technological progress in recent years. UUVs can accomplish important missions, such as marine surveying, plane crash searches, etc. [1,2,3,4]. The future development of UUVs will follow two important trends. The first is that sailing trajectories will increase in length and depth. The second is that more intelligent equipment will be developed to achieve more complex missions. UUV navigation is essential because it is the foundation for completing the missions. This paper focuses on two challenges for UUV navigation. The first challenge involves solving issues that occur when UUVs are sailing in the polar region. The second is completing missions with a team of UUVs. The algorithm proposed in this paper can effectively solve the cooperative navigation problems of multi-UUVs in regard to communication delays in the polar region.

The severe natural environment and extreme navigation environment have caused many challenges to UUV missions in the polar region. Strapdown Inertial Navigation Systems (SINS) are the most widely used navigation device among the UUVs, since the SINS navigate UUVs autonomously. Multi-UUVs are faced with the same problems as single UUVs in the polar region, such as calculation overflow and error magnification, which cause the conventional north-oriented SINS to be invalid in the polar region [5]. The direction information and the orientation information cannot be distinguished, which causes the conventional wander-oriented SINS to be unavailable in the polar region [6]. The multipath effect in high latitude areas rapidly decreases the accuracy of the Global Positioning System (GPS), or even causes the failure of the GPS [7]. Therefore, it is necessary to propose a navigation algorithm that is available in the polar region and allows the cooperative sailing of multi-UUVs.

Teamwork is an essential part of work and school activities. It can improve work efficiency greatly, which is also true for UUVs. The intelligence development of a single UUV is limited, but it can be solved by multi-UUV cooperation. Multi-UUVs have a great advantage over a single UUV, especially in terms of time and efficiency. In general, there are two cooperative multi-UUV navigation types: the parallel type and the leader-follower type [8].

The cooperative navigation system, developed by the Virginia Tech research team, is a typical parallel UUV cooperative navigation system, in which the UUV sends information to its surrounding neighbors in the form of an acoustic broadcast [9]. The mobile baseline system, developed by the University of Porto [10], and the CADRE system (Cooperative Autonomy for Distributed Reconnaissance and Exploration System) developed by Massachusetts Institute of Technology (MIT) [11], are typical leader-follower UUV cooperative navigation systems. Using the leader-follower type, cooperative navigation can be achieved among multi-UUVs. There is no need for all the UUVs to be equipped with high-precision navigation devices. Therefore, the leader-follower UUV cooperative navigation system has a lower cost.

Many novel algorithms have been proposed to achieve the cooperative navigation of multi-robots in non-polar regions [12,13,14], but none of them are suitable for multi-UUVs in the polar region. Some researchers [15,16] proposed a leader-follower UUV cooperative navigation with acoustic communication time delay compensation as an approach. However, this approach is influenced by the rapid convergence of Earth meridians in the polar region, which can only be used in the non-polar region. Simultaneously, the filter is based on a standard Kalman filter (KF), which can only be used in dynamic linear system models. Compared to the cooperative navigation of multi-UUVs in non-polar regions, cooperative navigation of multi-UUVs in polar regions is still in the beginning stages, and few papers currently cover this aspect. The algorithm proposed in this paper aims to achieve cooperative navigation of multi-UUVs in the polar region, and a dynamic non-linear system model is considered. Based on the polar grid navigation algorithm and the leader-follower type, a polar cooperative navigation algorithm of multi-UUVs that considers communication delays is discussed in this paper. The leader UUV is equipped with high-precision navigation devices and the follower UUVs are equipped with low-precision navigation devices [17]. The leader UUV sends the information through underwater acoustic communication to the follower UUVs. In addition, the relative positions between the leader UUV and follower UUVs are determined by ultra-short baseline (USBL) acoustic positioning. Based on the information sent by the leader UUV and the relative position measured by the USBL, the follower UUVs can modify the navigation results from their own navigation devices through the filter method. Both the leader UUV and the follower UUVs can obtain accurate navigation results by using this polar cooperative navigation algorithm for multi-UUVs that considers communication delays.

To solve navigation problems of multi-UUVs in the polar region, a polar cooperative navigation algorithm for multi-UUVs that considers communication delays is proposed in this paper. The development of the polar cooperative navigation algorithm and modification of the adaptive Kalman filter (AKF) based on communication delays are the two main contributions of this paper. Based on this algorithm, cooperative navigation is achieved among the leader-follower UUVs team in the polar region. Although the follower UUVs are equipped with low-precision navigation devices, they can achieve high navigation accuracy with this algorithm. The following sections are arranged as follows. The formation configuration, acoustic communication delay and overall process of the polar cooperative navigation algorithm are discussed in Section 2. The error equations and polar cooperative navigation filter models are deduced in Section 3 and Section 4, respectively. The conventional AKF is modified in Section 5 with consideration to communication delays. The simulation and experiment results are presented in Section 6. Finally, Section 7 gives the conclusions.

## 2. Polar Cooperative Navigation System

Different formation configurations and acoustic communication modes will have different effects on the polar cooperative navigation system of multi-UUVs. Under the premise of ensuring navigation accuracy, it is our goal to reduce costs and improve efficiency as much as possible. This section will focus on the formation configuration, underwater acoustic communication and overall process of the polar cooperative navigation system.

### 2.1. Formation Configuration

**Figure 1:**
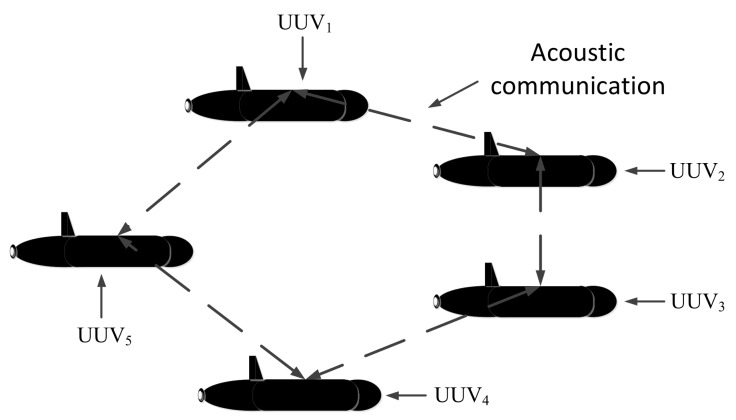
Unmanned Underwater Vehicles (UUV) cooperative navigation system—parallel UUV cooperative navigation system.

Typical formation configurations of multi-UUVs mainly include the parallel type and the leader-follower type. In the parallel type, all the UUVs are required to have the same configuration. This means that all the UUVs should be equipped with high-precision navigation devices, and each UUV communicates with its neighbor UUVs. The number of multi-UUVs in a parallel, polar cooperative navigation system is limited by the underwater acoustic communication bandwidth. A parallel cooperative navigation system of multi-UUVs is shown in Figure 1, which shows five UUVs as an example.

**Figure 2:**
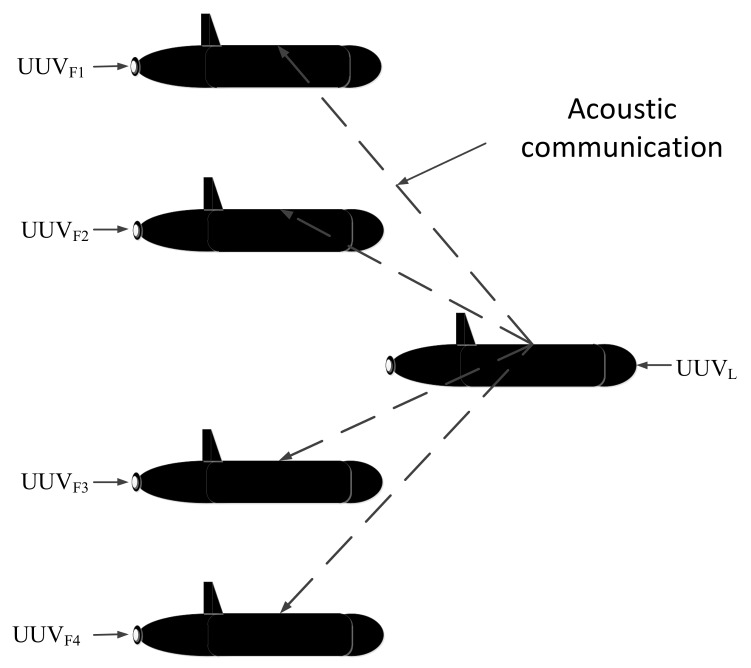
UUV cooperative navigation system—leader-follower UUV cooperative navigation system.

Unlike the parallel type, the configuration of each UUV in the leader-follower type can be different. The leader UUV is equipped with high-precision navigation devices, and the follower UUVs are equipped with low-precision navigation devices, which can reduce costs. In addition, the underwater acoustic communication is only required between the leader UUV and follower UUVs. There is no need for follower UUVs to communicate with each other. The limitations caused by underwater acoustic communication bandwidth are greatly reduced. A typical leader-follower cooperative navigation system usually includes a single leader or double leaders. A leader-follower cooperative navigation system of multi-UUVs is shown in Figure 2, which shows one leader UUV and four follower UUVs as an example. In Figure 2, UUV_L_ is the leader UUV, and UUV_F1_, UUV_F2_, UUV_F3_, UUV_F4_ are the follower UUVs.

From the viewpoint of the underwater acoustic communication bandwidth and navigation equipment costs, the leader-follower type is superior to the parallel type. The leader UUV is equipped with high-precision navigation devices, including SINS, OCTANS (an all-in-one gyrocompass and motion sensor for diverse challenging applications, produced by iXblue), Doppler Velocity Log (DVL), depth meters and USBL. The follower UUVs are equipped with low-precision navigation devices only, including SINS, DVL, depth meters and USBL, to reduce costs.

### 2.2. Underwater Acoustic Communication Delay

Due to the extreme natural and navigation environment underwater in the polar region, communication becomes one of the difficulties in polar cooperative navigation of multi-UUVs. As the radio signal cannot transmit under deep water, underwater acoustic communication becomes the first choice for polar cooperative navigation systems. Underwater acoustic transducers are most widely used in underwater acoustic communication. Information is transmitted through sound waves rather than radio waves. Thus, underwater acoustic communication is less efficient than radio communication.

The working principles of underwater acoustic communication can be described as follows: First, information is converted into an electrical signal by an electrical transmitter. Second, after digital processing by an encoder, the transducer converts the electrical signal into an acoustic signal. Third, the acoustic signal propagates through the medium of water and propagates the information to the receiving transducer. In this case, the acoustic signal is converted into an electrical signal. Finally, after the digital signal is deciphered by the decoder, the information is converted to sound, text or picture by the electrical receiver.

According to the characters of underwater acoustic communication, there will be underwater acoustic delays in the propagation of underwater acoustics. These underwater acoustic delays usually consist of an underwater acoustic detection delay and an underwater communication delay. The underwater acoustic detection delay mainly depends on the detection equipment itself, and its value is relatively fixed and small. The underwater acoustic communication delay is caused by the high complexity of signal processing and the limitations from underwater bandwidth and low transmission rate. There will be a communication delay when the leader UUV sends its information to the follower UUVs in the polar cooperative navigation of multi-UUVs. As the efficiency of the underwater acoustic equipment varies with time and the distance between the leader UUV and the follower UUVs, the underwater acoustic communication delay is a random value [18]. According to the above analysis, the underwater acoustic delay can be expressed as follows:

(1) $$N_{i}=\Delta t+d/C+\delta t,$$

where Δt is the detection delay; d is the distance between the leader UUV and the follower UUV; C is the velocity of the underwater acoustic communication; and δt is the random part of the underwater acoustic communication delay.

### 2.3. Overall Process of the Polar Cooperative Navigation Algorithm

**Figure 3:**
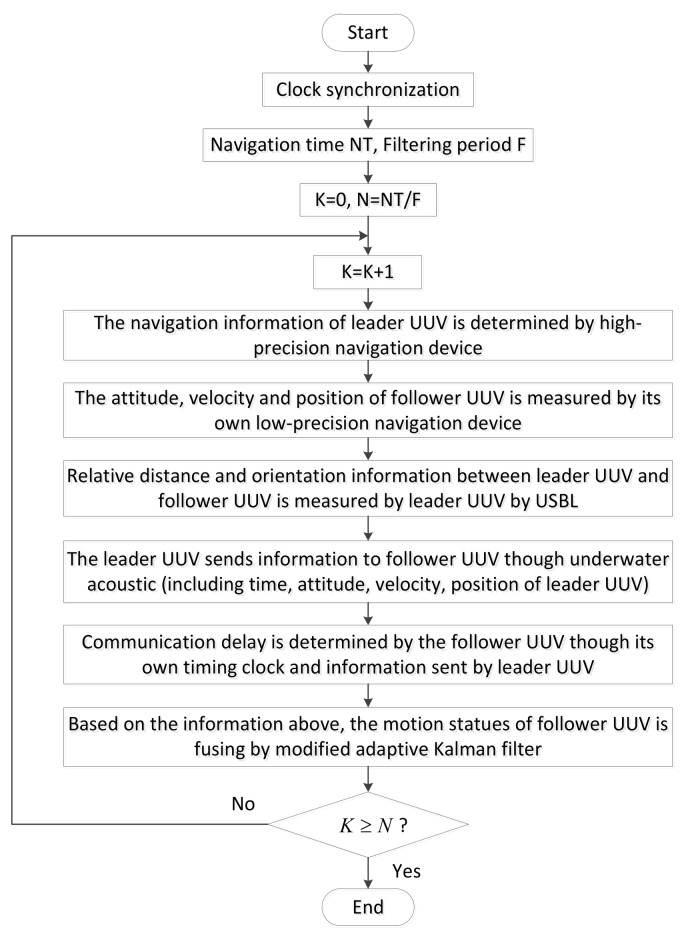
Overall process of the polar cooperative navigation algorithm.

Based on the above analysis, the leader-follower type is superior to the parallel type of navigation system. Like the formation configuration shown in Figure 2, the leader UUV is equipped with high-precision navigation devices. To simplify the analysis and combine the characteristics of this article, the leader UUV errors are ignored. As both the leader UUV and follower UUVs are equipped with depth meters, the depth information from each UUV can be obtained accurately. In addition, the communication mode from the leader UUV to each follower UUV is the same, so the navigation between one leader UUV and one follower UUV is considered in this paper. The situation of multiple follower UUVs can be deduced by analogy. The overall process of the polar cooperative navigation algorithm can be expressed as shown in Figure 3.

Both the leader UUV and the follower UUVs are equipped with high-precision timing clocks. The clock synchronization is achieved before the multi-UUVs start their mission. The leader UUV sends information to the follower UUV through underwater acoustic communication, including the time, attitude, velocity, position of the leader UUV and the relative position between the leader UUV and follower UUV. The relative distance and orientation information between the leader UUV and follower UUV can be measured by the leader UUV through USBL. Through the combination of the information sent by the leader UUV, and information from its own navigation devices, the motion states of a follower UUV can be estimated accurately.

## 3. Error Equations of the Polar Cooperative Navigation Algorithm

In the leader-follower polar cooperative navigation system, the leader UUV is equipped with high-precision navigation devices, including a high-precision SINS, OCTANS, DVL, USBL and depth meter. Based on the polar grid navigation algorithm proposed in ref. [19], the leader UUV can accurately navigate in the polar region. Therefore, the attitude errors, velocity errors and position errors of the leader UUV are assumed to be zero. To save costs, the follower UUVs are equipped with low-precision navigation devices, including a low-precision SINS, DVL, USBL and depth meter. The navigation errors of the follower UUVs are bigger than those of the leader UUV, which cannot be ignored. The error equations of the follower UUV should be established based on the UUV characteristics and the environment of the polar region.

In the polar region, there will be calculation overflow and error magnification of the navigation in conventional north-oriented SINS. The direction information and the orientation information cannot be distinguished in conventional wander-oriented SINS. Therefore, conventional SINS is invalid in the polar region. The polar grid navigation algorithm proposed in [19] can solve these problems. Based on the polar grid navigation algorithm and the characteristics of a UUV, the attitude error equation, velocity error equation and position error equation of a follower UUV can be deduced as follows.

The relationships among the frames are important parts of the SINS. To simplify the description, the frames involved in this paper are briefly represented by the following symbols:

Inertial frame—i frame;
Navigation frame—n frame;
Body frame of UUV—b frame;
Geographic frame—g frame;
Earth centered earth fixed frame—e frame;
Grid frame—G frame;
Body frame of DVL—m frame;
Acoustic array frame of USBL—a frame.

### 3.1. Attitude Error Equation of a Follower UUV

Based on the polar grid navigation algorithm proposed in ref. [19] and in the G frame, the attitude differential equation of the follower UUV in ideal conditions and in actual conditions can be described by Equations (2) and (3), respectively.

(2) $${\dot{C}}_{b}^{G}=C_{b}^{G}\left[\omega_{ib}^{b}\times \right]-\left[\omega_{iG}^{G}\times \right]C_{b}^{G},$$

(3) $${\dot{\hat{C}}}_{b}^{G^{\prime }}={\hat{C}}_{b}^{G}\left[{\hat{\omega }}_{ib}^{b}\times \right]-\left[{\hat{\omega }}_{iG}^{G}\times \right]{\hat{C}}_{b}^{G},$$

where $C_{b}^{G}$ and ${\hat{C}}_{b}^{G}$ are the direction cosine matrices from the b frame to the G frame in ideal conditions and in actual conditions, respectively; $\omega_{ib}^{b}$ and ${\hat{\omega }}_{ib}^{b}$ are the rotational angular velocities from the i frame to the b frame in the i frame in ideal conditions and in actual conditions, respectively; and $\left[\omega_{ib}^{b}\times \right]$ is the anti-symmetric matrix of $\omega_{ib}^{b}$.

Subtracting Equation (2) from Equation (3), the attitude error equation of the follower UUV in the G frame can be expressed as follows:

(4) $${\dot{\phi }}^{G}=C_{\omega }{}^{-1}(-(\omega_{iG}^{G}\times )\phi^{G}+(C_{\omega ieR}+C_{\omega eGR})\delta R^{e}+C_{\omega eGv}\delta V^{G}-C_{b}^{G^{\prime }}\varepsilon^{b}),$$

where $\phi^{G}={\left[\phi_{x}^{G}\;\;\phi_{y}^{G}\;\;\phi_{z}^{G}\right]}^{T}$ is the misalignment angle; $\delta V^{G}={\left[\delta V_{x}^{G}\;\;\delta V_{y}^{G}\;\;\delta V_{z}^{G}\right]}^{T}$ is the velocity error; $\delta R^{e}={\left[\delta x\;\;\delta y\;\;\delta z\right]}^{T}$ is the position error; and $\varepsilon^{b}$ consists of gyro constant drifts, $\varepsilon_{c}^{b}$, and gyro random drifts, $\varepsilon_{w}^{b}$, that can be set as zero-mean Gaussian white noise.

(5) $$C_{\omega }=\left[\begin{array}{l} \cos\phi_{y}^{G}\;\;\;\;\;\;\;\;\;\;\;\;0\;\;\;\;\;\;\;\;\;\;\;-\sin\phi_{y}^{G}\cos\phi_{x}^{G}\\ \;\;\;\;0\;\;\;\;\;\;\;\;\;\;\;\;\;\;\;\;\;1\;\;\;\;\;\;\;\;\;\;\;\;\;\;\;\;\;\;\;\;\sin\phi_{x}^{G}\\ \sin\phi_{y}^{G}\;\;\;\;\;\;\;\;\;\;\;\;\;0\;\;\;\;\;\;\;\;\;\;\;\;\;\;\cos\phi_{y}^{G}\cos\phi_{x}^{G} \end{array}\right],$$

(6) $$\;C_{\omega ieR}=\frac{\omega_{ie}}{R_{eh}{\left(1-c^{2}Ls^{2}\lambda \right)}^{\frac{3}{2}}}\cdot \left[\begin{array}{l} 2c^{2}LsLs\lambda c\lambda \;\;\;\;\;\;-sL\left[c^{2}\lambda +s^{2}\lambda \cdot \left(s^{2}L-c^{2}L\right)\right]\;\;\;\;\;\;\;\;\;\;\;\;s^{2}LcLs\lambda \\ \;\;\;\;\;\;\;\;s^{2}L\;\;\;\;\;\;\;\;\;\;\;\;\;\;\;\;\;\;\;\;\;\;\;\;\;\;\;\;\;\;\;\;\;\;\;\;\;0\;\;\;\;\;\;\;\;\;\;\;\;\;\;\;\;\;\;\;\;\;\;\;\;\;\;\;\;\;\;\;\;\;\;\;\;\;\;\;-sLcLc\lambda \\ \;\;-sLcLc\lambda \;\;\;\;\;\;\;\;\;\;\;\;\;\;\;\;\;\;\;\;\;\;\;-sLcLs\lambda \;\;\;\;\;\;\;\;\;\;\;\;\;\;\;\;\;\;\;\;\;\;\;\;\;\;\;\;\;\;\;\;\;\;\;\;\;\;\;c^{2}L \end{array}\right],$$

(7) $$C_{\omega eGR}=\frac{1}{R_{eh}^{2}}\cdot \left[\begin{array}{l} \;\;\;\;v_{GN}cLc\lambda \;\;\;\;\;\;\;\;\;\;\;\;\;\;\;\;\;\;\;\;\;v_{GN}cLs\lambda \;\;\;\;\;\;\;\;\;\;\;\;\;\;\;\;\;\;\;\;\;\;\;\;\;v_{GN}sL\\ -v_{GE}cLc\lambda \;\;\;\;\;\;\;\;\;\;\;\;\;\;\;\;\;\;-v_{GE}cLs\lambda \;\;\;\;\;\;\;\;\;\;\;\;\;\;\;\;\;\;\;\;\;\;\;-v_{GE}sL\\ \;\frac{2\;v_{GN}\;\;c^{2}Ls\lambda c\lambda }{{\left(1-c^{2}Ls^{2}\lambda \right)}^{\frac{3}{2}}}\;\;\;\;\;\;\;\;\;-\;\;\;v_{GN}\sqrt{1-c^{2}Ls^{2}\lambda }\;\;\;\;\;\;\;\;\;\frac{2\;v_{GN}\;\;sLcLs\lambda }{{\left(1-c^{2}Ls^{2}\lambda \right)}^{\frac{3}{2}}} \end{array}\right],$$

where s(⋅) and c(⋅) represent sin(⋅) and cos(⋅), respectively. L and λ represent the latitude and longitude of the position, respectively.

### 3.2. Velocity Error Equation of a Follower UUV

Based on the G frame, the velocity differential equations of the follower UUV in ideal conditions and in actual conditions can be described with Equations (8) and (9), respectively.

(8) $${\dot{V}}^{G}=C_{b}^{G}f^{b}-\left(2\omega_{ie}^{G}+\omega_{eG}^{G}\right)\times V^{G}+g^{G},$$

(9) $${\dot{\hat{V}}}^{G}={\hat{C}}_{b}^{G}{\hat{f}}^{b}-\left(2{\hat{\omega }}_{ie}^{G}+{\hat{\omega }}_{eG}^{G}\right)\times {\hat{V}}^{G}+{\hat{g}}^{G},$$

where $V^{G}$ and ${\hat{V}}^{G}$ are the velocities of the follower UUV in ideal conditions and in actual conditions, respectively; $f^{b}$ and ${\hat{f}}^{b}$ are the special forces measured by the SINS in ideal conditions and in actual conditions, respectively; $g^{G}$ and ${\hat{g}}^{G}$ are the gravity accelerations in ideal conditions and in actual conditions, respectively.

Subtracting Equation (8) from Equation (9) and ignoring the second-order small terms, the velocity error equation of the follower UUV can be expressed as follows:

(10) $$\delta {\dot{V}}^{G}=f^{G}\times \phi^{G}+\left[V^{G}\times C_{\omega eGV}-\left(2\omega_{ie}^{G}+\omega_{eG}^{G}\right)\times \right]\;\cdot \delta V^{G}\;+\left[V^{G}\times \left(2C_{\omega ieR}+C_{\omega eGR}\right)\right]\delta R^{e}+C_{b}^{G}\nabla^{b},$$

where $\nabla^{b}$ is the accelerator bias, consisting of an accelerator constant bias, $\nabla_{c}^{b}$, and an accelerator random bias, $\nabla_{w}^{b}$, which can be set as zero-mean Gaussian white noise [20].

### 3.3. Position Error Equation of the Follower UUV

Due to the rapid convergence of the Earth’s meridians and the meridians converging in the poles, the conventional latitude and longitude representation is no longer applicable in the polar region. In the polar grid navigation algorithm, the coordinates in the e frame, $R^{e}\left(x,y,z\right)$, are used to describe the position of the UUV.

Based on the G frame, the position’s differential equation for the follower UUV in ideal conditions and in actual conditions can be described by Equations (11) and (12), respectively.

(11) $${\dot{R}}^{e}=C_{G}^{e}V^{G},$$

(12) $${\dot{\hat{R}}}^{e}={\hat{C}}_{G}^{e}{\hat{V}}^{G},$$

where $R^{e}$ and ${\hat{R}}^{e}$ are the positions of the follower UUV in ideal conditions and in actual conditions, respectively; $C_{G}^{e}$ and ${\hat{C}}_{G}^{e}$ are the direction cosine matrices from the G frame to the e frame in ideal conditions and in actual conditions, respectively.

By subtracting Equation (11) from Equation (12), the position error equation of the follower UUV can be described as follows:

(13) $$\delta {\dot{R}}^{e}=C_{G}^{e}\delta V^{G}-C_{G}^{e}\left(V^{G}\times \right)C_{\beta R}\delta R^{e},$$

where

(14) $$C_{\beta R}=\frac{1}{R_{eh}}\left[\begin{array}{l} \;\;\frac{sL}{\sqrt{1-c^{2}Ls^{2}\lambda }}\;\;\;\;\;\;\;\;\;\;\;\;\;\;\;0\;\;\;\;\;\;\;\;\;\;\;\;\;\;\;\;\frac{-c\lambda cL}{\sqrt{1-c^{2}Ls^{2}\lambda }}\\ \frac{s\lambda c\lambda c^{2}L}{\sqrt{1-c^{2}Ls^{2}\lambda }}\;\;\;\;\;\;\sqrt{1-c^{2}Ls^{2}\lambda }\;\;\;\;\;\;\;\frac{-s\lambda sLcL}{\sqrt{1-c^{2}Ls^{2}\lambda }}\\ \frac{cLsLs\lambda }{1-c^{2}Ls^{2}\lambda }\;\;\;\;\;\;\;\;\;\;\;\;\;\;\;\;\;\;\;0\;\;\;\;\;\;\;\;\;\;\;\;\;\;-\;\frac{c^{2}Ls\lambda c\lambda }{1-c^{2}Ls^{2}\lambda } \end{array}\right],$$

### 3.4. Error Model of DVL in the Follower UUV

Because of the accumulative errors in the SINS and the low-precision navigation devices, there will be cumulative errors in the follower UUV. According to the Doppler Effect, the DVL can measure the velocity of the UUV. With the help of the DVL, the follower UUV can correct its velocity to improve the navigation accuracy. However, there are installation errors, scale factor errors, and random measurement errors affecting the accuracy of the DVL, because of the characteristics of the DVL. The output of the DVL can be described in the m frame and the G frame as Equations (15) and (16):

(15) $${\hat{V}}_{d}^{m}=\left(1+\delta K_{d}\right)V_{d}^{m}+\delta V_{d}^{m}+v_{d}^{m},$$

(16) $${\hat{V}}_{d}^{G}=V_{d}^{G}+v_{d}^{G}=C_{b}^{G}C_{m}^{b}V_{d}^{m}=C_{b}^{G}\left[\left({\hat{V}}_{d}^{m}-\delta V_{d}^{m}\right)/\left(1+\delta K_{d}\right)\right],$$

where ${\hat{V}}_{d}^{m}$ and $V_{d}^{m}$ are the actual velocity and ideal velocity, respectively; $C_{m}^{b}$ is the direction cosine matrix from the m frame to the b frame, which is approximately equal to $C_{m}^{b}=I$, since the installation error is small enough to be neglected; the scale factor and the random velocity error can be expressed by a random constant and the one-order Markov process as follows:

(17) $$\left\{ \begin{array}{l} \delta {\dot{K}}_{d}=0\\ \delta {\dot{V}}_{d}^{m}=-\delta V_{d}^{m}/\tau_{v}+w_{v} \end{array}\right.,$$

where $\tau_{v}$ is the correlation time and $w_{v}$ is the white noise.

### 3.5. Error Model of USBL

The USBL can obtain the relative position between the leader UUV and a follower UUV through the time that the acoustic signal arrives at the acoustic element. Since the size of the USBL acoustic array is particularly small, it is easy to choose a position on the carrier with low noise. Therefore, the application of the USBL is flexible and convenient.

The USBL is mainly composed of a transducer, an acoustic emission array, a transponder and an acoustic receiving array. The acoustic emission array and acoustic receiving array are fixed in the transducer to form the acoustic array. The mutual positions between the acoustic elements constituting the acoustic array have been precisely determined. The transponder is installed on the follower UUV. The phase comparison method is used in this system. The orientation of the transponder in the acoustic array frame is obtained by measuring the phase difference of the acoustic element. The relative distance between the leader UUV and the follower UUV is calculated by the propagation time of the acoustic signal in water. According to the above information, the relative position between the leader UUV and follower UUV can be determined.

(18) $$R^{2}=X_{a}^{2}+Y_{a}^{2}+h^{2},$$

(19) $$X_{a}^{2}=R^{2}\cos^{2}\theta_{mx},$$

(20) $$Y_{a}^{2}=R^{2}\cos^{2}\theta_{my},$$

where R is the distance from the acoustic emission array to the acoustic receiving array; $X_{a}$ and $Y_{a}$ are the coordinates of the follower UUV in the acoustic array frame; $\theta_{mx}$ and $\theta_{my}$ can be measured by the USBL; $\theta_{mx}$ is the angle between the acoustic ray and the $X_{a}$ axis; $\theta_{my}$ is the angle between the acoustic ray and the $Y_{a}$ axis; h is the relative depth difference between the leader UUV and the follower UUV. In addition, the depth information regarding the leader UUV and the follower UUV can be obtained from their own depth meters, respectively.

From Equations (18)–(20), $X_{a}$ and $Y_{a}$ can be obtained as follows:

(21) $$X_{a}=\frac{h\cos\theta_{mx}}{\sqrt{1-\cos^{2}\theta_{mx}-\cos^{2}\theta_{my}}},$$

(22) $$Y_{a}=\frac{h\cos\theta_{my}}{\sqrt{1-\cos^{2}\theta_{mx}-\cos^{2}\theta_{my}}},$$

The main factors that affect the accuracy of the USBL are the error of the system, a measurement error for the marine environmental parameters and an acoustic array installation error. To simplify the analysis and highlight the subject of this paper, these errors are regarded as zero-mean Gaussian white noise. The error equation of the USBL in the e frame can be described as follows:

(23) $$\delta {\dot{R}}_{a}^{e}=C_{a}^{e}\delta {\dot{R}}^{a}=C_{a}^{e}\beta^{a},$$

where $\beta^{a}$ is zero-mean Gaussian white noise.

## 4. Filter Models of the Follower UUV in the Polar Cooperative Navigation Algorithm

Filter models of the follower UUV can be established based on the analysis in Section 3. The filter models consist of a dynamic model and an observation model. Details about the filter models are described as follows.

### 4.1. Dynamic Model

The states in the follower UUV are SINS states, DVL states and USBL states, which can be described as:

$$X={\left[{\left(\phi^{G}\right)}^{T}\;{\left(\delta V^{G}\right)}^{T}\;\;{\left(\delta R^{e}\right)}^{T}\;{\left(\varepsilon_{c}^{b}\right)}^{T}\;\;{\left(\nabla_{c}^{b}\right)}^{T}\;\;\;{\left(\delta K_{d}\right)}^{T}\;\;{\left(\delta V_{d}^{m}\right)}^{T}\;{\left(\beta^{a}\right)}^{T}\right]}^{T}$$

Based on the attitude error equation, velocity error equation, and position error equation of the SINS (Equations (4), (10) and (13)) and the error models of the DVL and USBL (Equations (17) and (23)), the dynamic model of the follower UUV can be described as follows:

(24) $$\{\begin{array}{l} {\dot{\phi }}^{G}=C_{\omega }{}^{-1}[-(\omega_{iG}^{G}\times )\phi^{G}+(C_{\omega ieR}+C_{\omega eGR})\delta R^{e}+C_{\omega eGv}\delta V^{G}-C_{b}^{G^{\prime }}\varepsilon^{b}]\\ \begin{array}{l} \delta {\dot{V}}^{G}=f^{G}\times \phi^{G}+[V^{G}\times C_{\omega eGv}\;\;-\left(2\omega_{ie}^{G}+\omega_{eG}^{G}\right)\times ]\cdot \delta V^{G}\;+\left[V^{G}\times \left(2C_{\omega ieR}+C_{\omega eGR}\right)\right]\delta R^{e}+C_{b}^{G}\nabla^{b}\\ \delta {\dot{R}}^{e}=C_{G}^{e}\delta V^{G}-C_{G}^{e}\left(V^{G}\times \right)C_{\varphi R}\delta R^{e} \end{array}\\ {\dot{\varepsilon }}^{b}\;=\;0\\ \begin{array}{l} {\dot{\nabla }}^{b}\;=\;0\\ \delta {\dot{K}}_{d}=0\\ \delta {\dot{V}}_{d}^{m}=-\delta V_{d}^{m}/\tau_{V}+w_{V}\\ {\dot{\beta }}^{a}=0 \end{array} \end{array},$$

This dynamic model can be expressed in the form of a vector as:

(25) $$\dot{X}=AX+BW,$$

where A and B are the system matrix and control matrix, respectively. W is the system noise matrix. A, B and W can be expressed as follows:

$$A=\left[\begin{array}{l} A_{15\times 15}^{SINS}\;\;\;\;\;\;\;\;\;\;\;\;\;\;\;\;\;0_{15\times 6}\;\;\;\;\;\;\;\;\;\;\;\;\;\;\;\;\;0_{15\times 3}\\ 0_{6\times 15}\;\;\;\;\;\;\;\;\;\;\;\;\;\;\;\;\;A_{6\times 6}^{DVL}\;\;\;\;\;\;\;\;\;\;\;\;\;\;\;\;\;\;\;\;0_{6\times 6}\;\\ 0_{3\times 15}\;\;\;\;\;\;\;\;\;\;\;\;\;\;\;\;\;\;\;\;0_{3\times 6}\;\;\;\;\;\;\;\;\;\;\;\;\;\;\;\;\;A_{3\times 3}^{USBL} \end{array}\right],$$

$$B={\left[\begin{array}{l} B_{1}\;\;\;\;\;\;\;0_{3\times 3}\;\;\;\;\;\;0_{3\times 3}\\ 0_{3\times 3}\;\;\;\;\;B_{2}\;\;\;\;\;\;\;0_{3\times 3}\\ 0_{12\times 3}\;\;\;0_{12\times 3}\;\;\;0_{12\times 3}\\ 0_{3\times 3}\;\;\;\;\;0_{3\times 3}\;\;\;\;\;I_{3\times 3}\\ 0_{3\times 3}\;\;\;\;\;0_{3\times 3}\;\;\;\;\;0_{3\times 3} \end{array}\right]}^{T},\;A_{15\times 15}^{SINS}=\left[\begin{array}{l} A_{1}\;\;\;\;\;\;\;\;\;A_{2}\;\;\;\;\;\;\;\;\;A_{3}\;\;\;\;\;\;\;\;\;A_{4}\;\;\;\;\;\;\;\;\;0_{3\times 3}\\ A_{5}\;\;\;\;\;\;\;\;\;A_{6}\;\;\;\;\;\;\;\;\;A_{7}\;\;\;\;\;\;\;\;\;0_{3\times 3}\;\;\;\;\;\;\;\;\;A_{8}\\ 0_{3\times 3}\;\;\;\;\;\;A_{9}\;\;\;\;\;\;\;\;\;A_{10}\;\;\;\;\;\;\;\;0_{3\times 3}\;\;\;\;\;\;\;\;\;0_{3\times 3}\\ 0_{3\times 3}\;\;\;\;\;\;\;\;0_{3\times 3}\;\;\;\;\;\;\;0_{3\times 3}\;\;\;\;\;0_{3\times 3}\;\;\;\;\;\;\;\;0_{3\times 3}\\ 0_{3\times 3}\;\;\;\;\;\;\;\;0_{3\times 3}\;\;\;\;\;\;\;0_{3\times 3}\;\;\;\;\;0_{3\times 3}\;\;\;\;\;\;\;\;0_{3\times 3} \end{array}\right],\;A_{6\times 6}^{DVL}=\left[\begin{array}{l} 0_{3\times 3}\;\;\;\;\;\;\;\;\;\;\;\;\;\;0_{3\times 3}\;\\ \;0_{3\times 3}\;\;\;\;\;\;\;\;\;\;\;\;A_{11} \end{array}\right],\;A_{3\times 3}^{USBL}=\left[0_{3\times 3}\right],\;W={\left[{\left(\varepsilon_{r}^{b}\right)}^{T}\;\;\;\;\;\;\;\;{\left(\nabla_{r}^{G}\right)}^{T}\;\;\;\;\;\;\;\;{\left(w_{v}\right)}^{T}\right]}^{T}.$$

where $A_{1}=C_{\omega }{}^{-1}\;(-(\omega_{iG}^{G}\times ))$, $A_{2}=C_{\omega }{}^{-1}C_{\omega eGv}\;$, $A_{3}=C_{\omega }{}^{-1}(C_{\omega ieR}+C_{\omega eGR})$, $A_{4}=-C_{\omega }{}^{-1}C_{b}^{G^{\prime }}$, $A_{5}=\left(f^{G}\times \right)$, $A_{6}=\left[V^{G}\times C_{\omega eGv}-\left(2\omega_{ie}^{G}+\omega_{eG}^{G}\right)\times \right]$, $A_{7}=\;\;V^{G}\times \left(2C_{\omega ieR}+C_{\omega eGR}\right)$, $A_{8}=C_{b}^{G}$, $A_{9}=C_{G}^{e}$, $A_{10}=-C_{G}^{e}\left(V^{G}\times \right)C_{\beta R}$, $A_{11}=-1/\tau_{v}\cdot I_{3\times 3}$, $B_{1}=C_{\omega }{}^{-1}C_{b}^{G^{\prime }}$, $B_{2}=C_{b}^{G}$.

### 4.2. Observation Model

The SINS equipped in the follower UUV has low precision. There are accumulated errors in the follower UUV, which cannot be ignored. Because the leader UUV is equipped with high-precision navigation devices, the information from the leader UUV can be used to correct the errors of the follower UUV. Based on the information sent from the leader UUV and the measurement results from the USBL, accurate position information for the follower UUV can be obtained. Therefore, the position error is chosen as one of the observation states. In addition, the accurate velocity information regarding the follower UUV can be provided by the DVL. Therefore, the velocity of the follower UUV calculated from the SINS can be corrected by the DVL. The other observation state is the velocity error. Based on the analysis above, the observation states can be expressed as:

$$Z={\left[\begin{array}{cc} {\left(\delta V^{G}\right)}^{T} & {\left(\delta R^{e}\right)}^{T} \end{array}\right]}^{T}$$

The observation model can be described as follows:

(26) $$\dot{Z}=HX+V,$$

where H and V are the observation matrix and measurement noise vector, respectively. V is regarded as zero-mean Gaussian white noise and H can be expressed as follows:

$$H={\left[\begin{array}{l} I_{3\times 3}\;\;\;\;\;\;\;\;\;0_{3\times 3}\;\;\;\;\;\;\;\;\;\;0_{3\times 3}\;\;\;\;\;\;\;\;\;\;\;0_{3\times 15}\\ 0_{3\times 3}\;\;\;\;\;\;\;\;0_{3\times 3}\;\;\;\;\;\;\;\;\;\;I_{3\times 3}\;\;\;\;\;\;\;\;\;\;\;\;0_{3\times 15} \end{array}\right]}^{T}$$

## 5. Modified Adaptive Kalman Filter Considering Communication Delays

**Figure 4:**
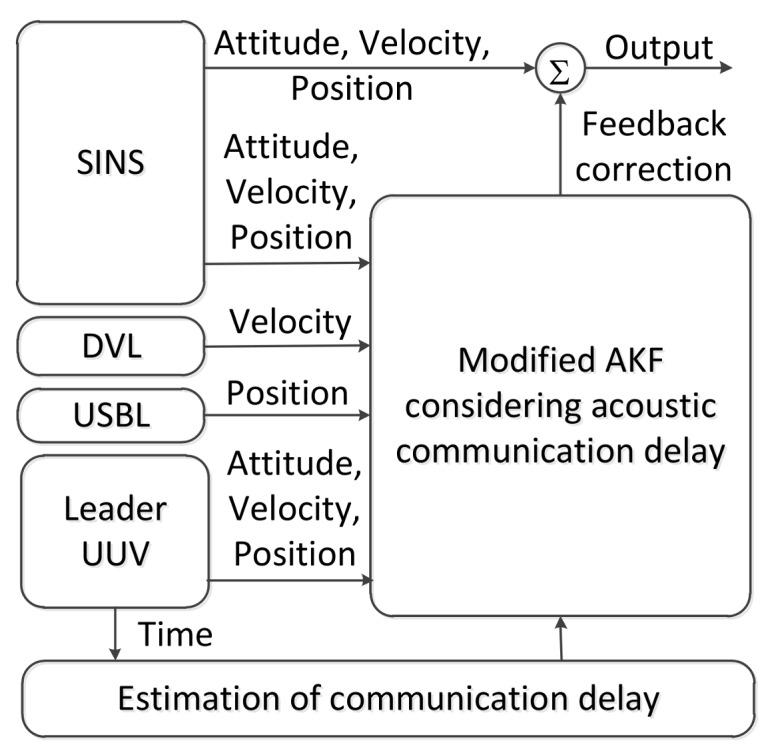
The schematic diagram of polar cooperative navigation of multi-UUVs considering communication delays.

According to the characteristics of polar cooperative navigation, there is a communication delay in the process of multi-source information fusion. Based on the acoustic communication delay’s characteristics and the role of underwater acoustic communication in the whole system, a system model of underwater acoustic delay can be constructed. In conventional AKF, the estimation results of the states are corrected according to real-time measurement information. Thus, conventional AKF which does not consider communication delay is not suitable for polar cooperative navigation of multi-UUVs. The schematic diagram of polar cooperative navigation of multi-UUVs considering communication delays is expressed in Figure 4.

The estimation processes of attitude, velocity and position in the follower UUV are described in Figure 4. These states are provided by the SINS in the follower UUV. Because of its low precision, there are errors in these states. The DVL, USBL and the information sent from the leader UUV are used to correct these states. Due to the underwater acoustic communication delay, the feedback position received by the follower UUV at time k is not the position measured at time k. Conventional AKF corrects these states based on the real-time measured states. This method is not suitable for communication delay conditions. The conventional AKF is modified in this paper to consider underwater acoustic communication delays. The states of the follower UUV at time k are corrected based on the states received by the follower UUV at time k. In addition, these states are not measured at time k. More accurate results can be obtained from the modified AKF.

The discrete expressions of the filter model and observation model can be expressed as follows:

(27) $$\dot{X}=\Phi_{k,k-1}X+\Gamma_{k,k-1}W,$$

(28) $$\dot{Z}=H_{k}X+V,$$

where the discrete expressions of A, B and H are $\Phi_{k,k-1}$, $\Gamma_{k,k-1}$ and $H_{k}$, respectively. Considering the characteristics of the application environment, the quantitative analysis is discussed in the following section. The conventional AKF is modified, as follows, to adapt for use in the polar cooperative navigation of multi-UUVs considering communication delays.

### 5.1. Conventional Adaptive Kalman Filter

Conventional AKF mainly consists of state updates (prediction) and measurement updates (correction) [21,22]. The states of this moment are estimated by the comprehensive states obtained at the last moment. Because of errors, these states need to be corrected based on the measurement results. Then the estimation result for the states can be obtained. The complete formula for the conventional AKF can be expressed by Equations (29)–(38).

(29) $$X_{k,k-1}=\Phi_{k,k-1}{\hat{X}}_{k-1}+{\hat{q}}_{k},$$

(30) $$P_{k,k-1}=\Phi_{k,k-1}P_{k-1}\Phi_{k,k-1}^{T}+\Gamma_{k,k-1}{\hat{Q}}_{k-1}\Gamma_{k,k-1}^{T},$$

(31) $$v_{k}=Z_{k}-H_{k}X_{k/k-1}-{\hat{r}}_{k},$$

(32) $$K_{k}=P_{k,k-1}H_{k}^{T}{\left[H_{k}P_{k/k-1}H_{k}^{T}+{\hat{R}}_{k}\right]}^{-1},$$

(33) $${\hat{X}}_{k}={\hat{X}}_{k/k-1}+K_{k}v_{k},$$

(34) $$P_{k}=(I-K_{k}H_{k})P_{k/k-1},$$

(35) $${\hat{q}}_{k+1}=(1-d_{k}){\hat{q}}_{k}+d_{k}(X_{k+1}-X_{k+1,k}),$$

(36) $${\hat{Q}}_{k+1}=(1-d_{k}){\hat{Q}}_{k}+d_{k}[K_{k+1}V_{k+1}{\left(K_{k+1}V_{k+1}\right)}^{T}+P_{k+1}-\Phi_{k+1,k}P_{k}{\hat{X}}_{k}\Phi_{k+1,k}^{T}],$$

(37) $${\hat{r}}_{k+1}=(1-d_{k}){\hat{r}}_{k}+d_{k}(Z_{k+1}-H_{k+1,k}X_{k+1,k}),$$

(38) $${\hat{R}}_{k+1}=(1-d_{k}){\hat{R}}_{k}+d_{k}[V_{k+1}V_{k+1}^{T}-H_{k+1}P_{k+1,k}H_{k+1}^{T}],$$

where P is the covariance of the states; the mean and the covariance of the system noise matrix, W, are ${\hat{q}}_{k}$ and ${\hat{Q}}_{k}$, respectively; ${\hat{r}}_{k}$ and ${\hat{R}}_{k}$ are the mean and the covariance of the measurement noise matrix, V; $d_{k}=(1-b)/(1-b^{k})$ and 0<b<1 are the forgetting factors.

### 5.2. Underwater Acoustic Communication Delay in AKF

Due to the underwater acoustic communication delay, the states of the follower UUV cannot be corrected by the measurement results at the current time. To meet the needs of the multi-UUVs cooperative navigation algorithm considering communication delays for use in the polar region, the conventional AKF needs to be modified. The underwater acoustic communication delay is a major factor in improving the accuracy of the conventional AKF. The analysis and special representation of underwater acoustic communication delays are discussed in the following text.

**Figure 5:**
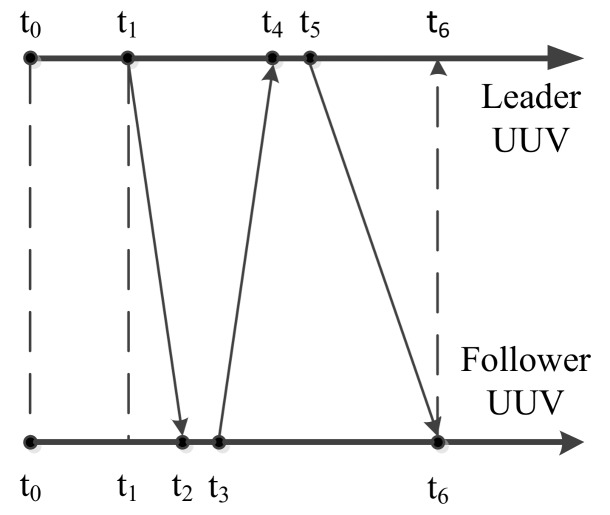
Time axes of the leader UUV and follower UUV.

According to the analysis in Section 2, the underwater acoustic communication delay is a random value. The acoustic communication delay can be easily understood by comparing the time axes of the leader UUV and the follower UUV. The time axes of the leader UUV and the follower UUV are shown as Figure 5.

As shown in Figure 5, it is assumed that the leader UUV’s time and the follower UUV’s time are the same. The initial time of the leader UUV, *t*_0_, is also the initial time of the follower UUV. The leader UUV sends a detection signal through USBL to the follower UUV at *t*_1_. At the same time, information about the states of the leader UUV is self-recorded. Because of the underwater acoustic communication delay, this detection signal will be received by the follower UUV at *t*_2_. After a short reaction time, the follower UUV sends a confirming signal to the leader UUV through USBL at *t*_3_. This confirming signal is received by the leader UUV at *t*_4_. The leader UUV calculates the relative positions of the leader UUV and the follower UUV. Then, the relative position and the information of the leader UUV at *t*_1_ are sent to the follower UUV at *t*_5_. After broadcasting through the water, this information is received by the follower UUV at *t*_6_. The underwater acoustic delay can be described as follows [14,15].

(39) $$\Delta t_{1}=t_{2}-t_{1},$$

(40) $$\;\Delta t_{2}=t_{3}-t_{2},$$

(41) $$\Delta t_{3}=t_{4}-t_{3},$$

(42) $$\Delta t_{4}=t_{5}-t_{4},$$

(43) $$\Delta t_{5}=t_{6}-t_{5},$$

(44) $$\Delta t_{6}=t_{6}-t_{1},$$

where $\Delta t_{1}$ is the transmission time of the acoustic signal from the leader UUV to the follower UUV; $\Delta t_{2}$ is the reaction time for the follower UUV to respond to the leader UUV through the USBL; $\Delta t_{3}$ is the transmission time for the acoustic signal from the follower UUV to the leader UUV; $\Delta t_{4}$ is the calculation time for the leader UUV to obtain the relative position between the leader UUV and the follower UUV through USBL; $\Delta t_{5}$ is the transmission time for the acoustic signal from the leader UUV to the follower UUV; $\Delta t_{6}$ is the total underwater acoustic delay during a measurement cycle.

The underwater acoustic delay consists of the underwater acoustic detection delay and the underwater acoustic communication delay. During the measurement cycle, both $\Delta t_{2}$ and $\Delta t_{4}$ are underwater acoustic detection delays. $\Delta t_{1}$, $\Delta t_{3}$ and $\Delta t_{5}$ are the acoustic communication delays. Compared with the underwater acoustic communication delay, the value of the underwater acoustic detection delay is relatively fixed and small. These underwater acoustic detection delays are associated with the USBL’s performance, and they can be reduced by improving its performance. To simplify the analysis of this paper, the acoustic detection delays are ignored. Both the underwater acoustic detection delay and the underwater acoustic communication delay are regarded as the underwater acoustic communication delay.

To describe the underwater acoustic communication delay better, it can be quantified. Since time synchronization is achieved between the leader UUV and the follower UUV and the underwater acoustic communication delay is a limited value, the underwater acoustic communication delay can be expressed versus time. Thus, the acoustic communication delay can be quantized. After quantification, the underwater acoustic communication delay can be expressed as follows:

(45) t=iT,

(46)  i∈{1,2,3,4,⋯,D},

where t is the underwater acoustic communication delay; T is the discretized unit time; i is an integer that is associated with the acoustic communication delay and D is an integer.

For example, t=2T and i=2. This means that the states are obtained at time k, while the observations are measured at time k−2. Replaced by a general representation, the observation at time k−i is used to correct the states at time k.

### 5.3. Modified Adaptive Kalman Filter Considering Communication Delays

Considering the existence of underwater acoustic communication delays, the information that the follower UUV receives from the leader UUV at time k is not the observation at time k. It is measured at time k−i [14,15]. Therefore, there will be errors when using conventional AKF. The states at time k are corrected by observations measured at time k in conventional AKF. However, in this paper, the states at time k need to be corrected by observations measured at time k−i. According to the analysis above, the conventional AKF is modified to adapt to the polar cooperative navigation of multi-UUVs with consideration of communication delays. After an iterative calculation has been completed i times, Equation (27) can be expressed as follows:

(47) $$X_{k}=\Phi_{k,k-i}X_{k-i}+\Phi_{k,k-i+1}\Gamma_{k,k-i+1}W_{k-i+1}+\cdots +\Gamma_{k,k-1}W_{k-1},$$

(48) $${\dot{\phi }}^{G}=C_{\omega }{}^{-1}\omega_{GG^{\prime }}^{G^{\prime }}=C_{\omega }{}^{-1}\left(\left(\phi^{G}\times \right)\omega_{iG}^{G}+\delta \omega_{iG}^{G}-C_{b}^{G^{\prime }}\delta \omega_{ib}^{b}\right),$$

Based on Equation (47), the states at time *k* − *i* can be described as follows:

(49) $$X_{k-i}=\Phi_{k,k-i}^{-1}X_{k}-\Phi_{k,k-i}^{-1}\Phi_{k,k-i+1}\Gamma_{k,k-i+1}W_{k-i+1}-\cdots -\Phi_{k,k-i}^{-1}\Gamma_{k,k-1}W_{k-1},$$

By substituting Equation (49) into (28), the observation model can be modified as follows:

(50) $$Z_{k-i}=H_{k-i}\Phi_{k,k-i}^{-1}X_{k}+{\bar{V}}_{k-i},$$

(51) $$\;{\bar{V}}_{k-i}=-H_{k-i}\Phi_{k,k-i}^{-1}\Phi_{k,k-i+1}\Gamma_{k,k-i+1}W_{k-i+1}\;-\cdots -H_{k-i}\Phi_{k,k-i}^{-1}\Gamma_{k,k-1}W_{k-1}+V_{k-i},$$

Therefore, the system model and observation model of the modified AKF adapted to polar cooperative navigation of multi-UUVs considering communication delays can be expressed as follows:

(52) $$X_{k}=\Phi_{k,k-1}X_{k-1}+\Gamma_{k,k-1}W_{k-1},$$

(53) $$Z_{k-i}=H\Phi_{k,k-i}^{-1}X_{k}+{\bar{V}}_{k-i},$$

To maintain the positive definiteness of the covariance of system noise matrix and measurement noise matrix as well as to simplify the system, Equations (36) and (38) in the conventional AKF are modified as follows:

(54) $${\hat{Q}}_{k+1}=(1-d_{k}){\hat{Q}}_{k}+d_{k}[K_{k+1}v_{k+1}{\left(K_{k+1}v_{k+1}\right)}^{T}+P_{k+1}],$$

(55) $${\hat{R}}_{k+1}=(1-d_{k}){\hat{R}}_{k}+d_{k}[v_{k+1}v_{k+1}^{T}],$$

The complete formula of the modified AKF considering communication delays and is adapted for the polar cooperative navigation of multi-UUVs can be expressed as follows:

(56) $$X_{k,k-1}=\Phi_{k,k-1}{\hat{X}}_{k-1}+{\hat{q}}_{k},$$

(57) $$P_{k,k-1}=\Phi_{k,k-1}P_{k-1}\Phi_{k,k-1}^{T}+\Gamma_{k,k-1}{\hat{Q}}_{k-1}\Gamma_{k,k-1}^{T},$$

(58) $$v_{k}=Z_{k-i}-H\Phi_{k,k-i}^{-1}X_{k/k-1}-{\hat{r}}_{k},$$

(59) $$K_{k}=P_{k,k-1}{\left[H\Phi_{k,k-i}^{-1}\right]}^{T}\cdot {\left[H\Phi_{k,k-i}^{-1}P_{k/k-1}{\left(H\Phi_{k,k-i}^{-1}\right)}^{T}+{\hat{R}}_{k}\right]}^{-1},$$

(60) $${\hat{X}}_{k}={\hat{X}}_{k/k-1}+K_{k}v_{k},$$

(61) $$P_{k}=(I-K_{k}H\Phi_{k,k-i}^{-1})P_{k/k-1},$$

(62) $${\hat{q}}_{k+1}=(1-d_{k}){\hat{q}}_{k}+d_{k}(X_{k+1}-X_{k+1,k}),$$

(63) $$\;{\hat{Q}}_{k+1}=(1-d_{k}){\hat{Q}}_{k}+d_{k}[K_{k+1}v_{k+1}{\left(K_{k+1}v_{k+1}\right)}^{T}+P_{k+1}],$$

(64) $$\;{\hat{r}}_{k+1}=(1-d_{k}){\hat{r}}_{k}+d_{k}(Z_{k+1}-H_{k+1,k}X_{k+1,k}),$$

(65) $$\;{\hat{R}}_{k+1}=(1-d_{k}){\hat{R}}_{k}+d_{k}[v_{k+1}v_{k+1}^{T}],$$

**Figure 6:**
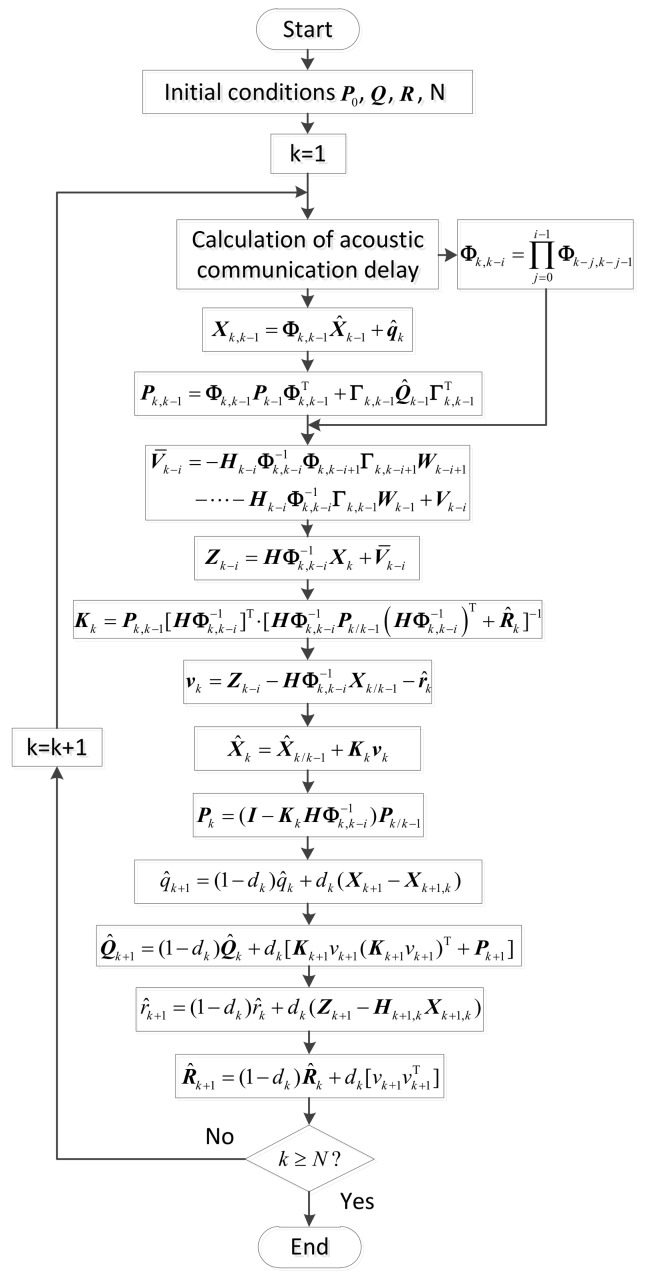
Flow chart of modified AKF considering acoustic communication delays.

To clearly express the modified AKF, the filter flow chart of the modified AKF used for the polar cooperative navigation of multi-UUVs considering communication delays is shown in Figure 6.

## 6. Results and Discussion

**Figure 7:**
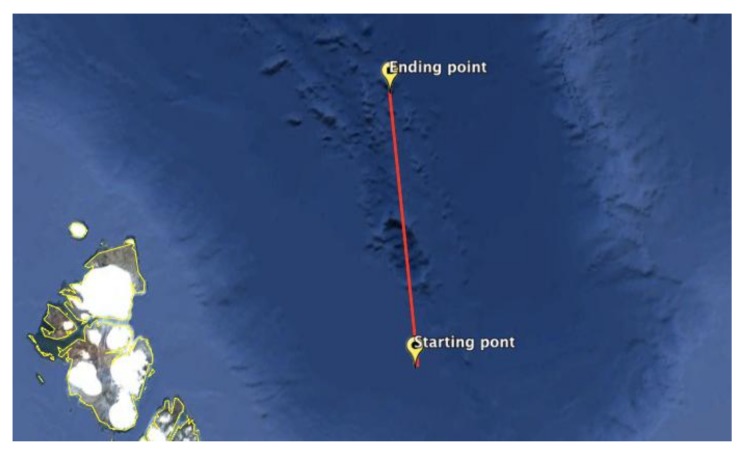
The sketch of the planned mission.

To verify the advantages of the proposed algorithm, a simulation and experiment were performed using the polar cooperative navigation algorithm of multi-UUVs considering communication delays. By comparing the results in different situations, the advantages of the proposed algorithm can be obtained. To simplify the analysis, the simulations and experiments met the limitations proposed in Section 2. Therefore, the leader-follower formation structure was adopted in this paper. The leader UUV was equipped with high-precision navigation devices and the navigation error could be ignored. The depth of both the leader UUV and follower UUV could be obtained accurately. Thus, the two-dimensional situation after projection is analyzed in this paper. The case of a single leader UUV and a single follower UUV is discussed in this paper. In addition, the timing of the UUVs was synchronized. The UUVs were ordered to finish a straight-line mission that is shown as Figure 7. The initial position of the UUV was (80° N, 120° E).

### 6.1. Simulation Results and Analyses

According to the conditions proposed above and the UUV characteristics, a simulation of the polar cooperative navigation algorithm of multi-UUVs considering communication delays was designed. The initial conditions and limitations were as follows:

System simulation conditions: the simulation time was 12 h; the filtering period was 0.1 s. Initial conditions of UUV: the initial position of UUV consisted of latitude, L, and longitude, λ; L was 80° and λ was 120°. The attitudes of the UUV, including pitch angle, roll angle, and yaw angle, were described by sine functions and the amplitude of these angles were 4°, 5° and 3°, respectively. The periods of these angles were 3 s 5 s, and 7 s, respectively. The initial phases of these angles were 0°, 0° and 0°, respectively. Initial conditions of SINS: the gyro drifts consisted of gyro constant drifts and gyro random drifts that were 0.03°/h and ${\left(0.001{}^{\circ}/h\right)}^{2}$, respectively. Similarly, the accelerometer bias consisted of the accelerometer constant bias and the accelerometer random bias, which were $1\times 10^{-6}g_{0}$ and ${\left(1\times 10^{-7}g_{0}\right)}^{2}$, respectively. Initial conditions of DVL: in the DVL, velocity random drifts were set as $\delta V_{dx}^{m}=\delta V_{dy}^{m}=\delta V_{dz}^{m}=0.005\;m/s$, and the correlation time was set as $\tau_{V}=5\;\min$, and the scale factor error was set as $\delta K_{d}{=10}^{-4}$.

**Figure 8:**
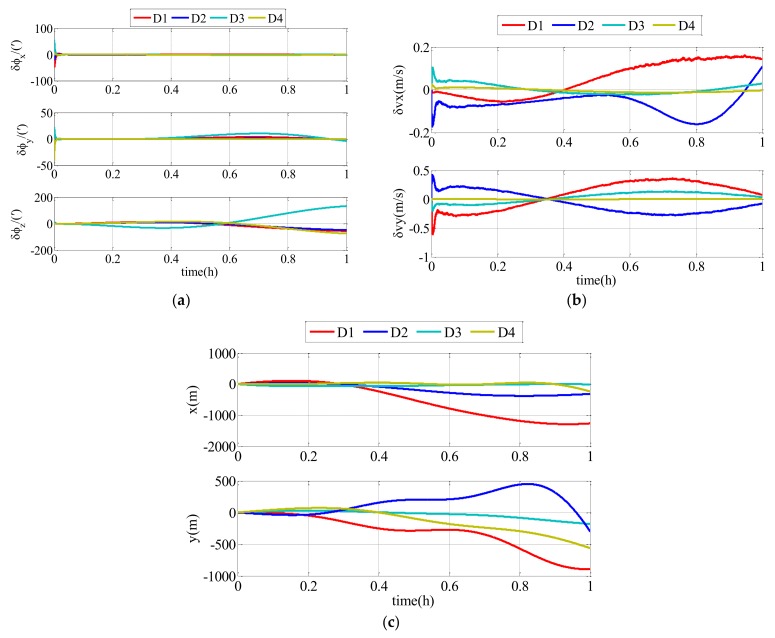
Estimation errors of conventional adaptive Kalman filter (AKF) with different ranges of random communication delay: (**a**) attitude estimation errors; (**b**) velocity estimation errors; (**c**) position estimation errors.

According to the conditions set above, the simulation results of the follower UUV, including attitude errors, velocity errors and position errors, can be expressed as described in the following text. There were two comparison simulations. In the first comparison simulation, different random communication delay ranges were set in the conventional AKF. The impact of communication delay on the conventional AKF is clearly expressed as Figure 8 and Table 1.

In this simulation, the simulation time was 1 h and D1≥D2≥D3≥D4. To clearly describe the impacts of the different ranges on the navigation accuracy of the follower UUV, the RMS errors of the attitude estimation errors, velocity estimation errors and position estimation errors are shown in Table 1. D1, D2, D3 and D4 represent the range integers of the communication delay and D1≥D2≥D3≥D4.

As shown in Figure 8 and Table 1, the communication delay had an impact on the navigation accuracy of the follower UUV. The errors were divergent with time flow, and the communication delay with a bigger range had a larger impact on the results. Therefore, the communication delay must be taken into consideration. The modified AKF described in this paper can effectively improve the navigation accuracy of the follower UUV.

**Figure 9:**
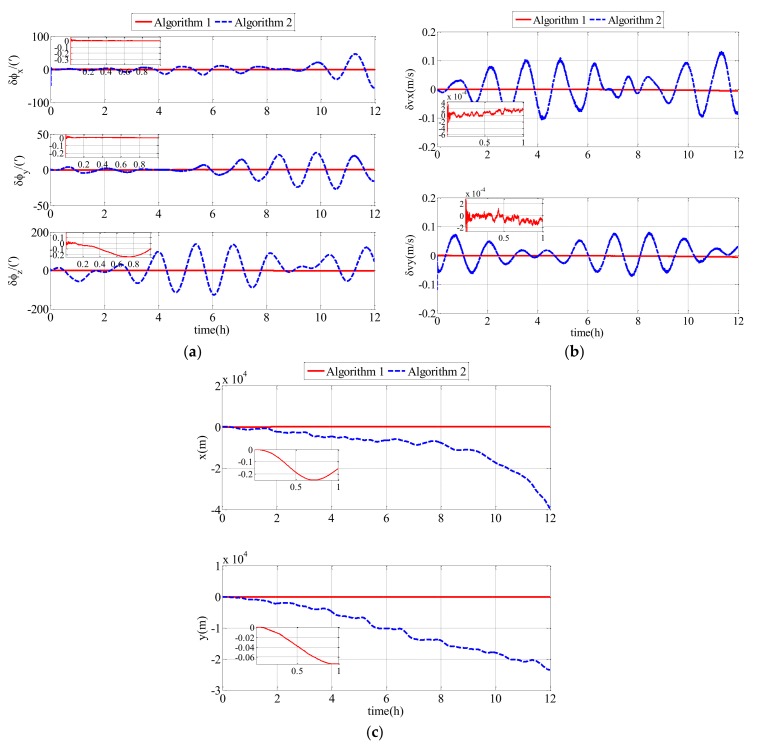
Estimation errors based on Algorithm 1 and Algorithm 2 in the simulation: (**a**) attitude estimation errors; (**b**) velocity estimation errors; (**c**) position estimation errors.

In the second comparison simulation, Algorithm 1 was compared with Algorithm 2. Algorithm 1 represents the polar cooperative navigation algorithm of multi-UUVs considering communication delays that is proposed in this paper. Algorithm 2 represents the polar cooperative navigation algorithm of multi-UUVs based on conventional AKF that does not consider communication delays. The estimation errors of the follower UUV, including attitude errors, velocity errors and position errors, are shown as Figure 9.

For a clearer description of the results obtained from the simulation, the RMS errors from attitude errors, velocity errors and position errors are shown in Table 2.

As shown in Figure 9 and Table 2, the proposed polar cooperative navigation algorithm of multi-UUVs considering communication delays was superior to the polar cooperative navigation algorithm based on conventional AKF. In the polar cooperative navigation algorithm of multi-UUVs considering communication delays, the estimation errors of attitude, velocity and position in the follower UUV converged quickly, and they were stable near zero.

### 6.2. Experiment Results and Analyses

The polar cooperative navigation algorithm of multi-UUVs considering communication delays has been discussed in this paper. Due to geographical constraints, the current experiment could be conducted in the polar region. Therefore, a semi-physical simulation experiment was performed, which means that the experiment was conducted in a non-polar region and the data measured during the experiment is used for the semi-physical simulation. According to the UUV characteristics, gyro drifts and accelerometer bias are natural characteristics of UUVs. They do not change in different locations. Therefore, the gyro drifts and accelerometer bias can be obtained from an experiment in the non-polar region. Practically-measured data and the simulated data comprise the experimental data. The experimental data is composed of the angular velocity, $\;{\hat{\omega }}_{ib}^{b}$, and special force, $\;{\hat{f}}^{b}$. The true angular velocity, $\;\omega_{ib}^{b}$, and gyro drifts, $\;\delta \omega_{ib}^{b}$, comprise the angular velocity, $\;{\hat{\omega }}_{ib}^{b}$. The true special force, $\;f^{b}$, and accelerometer bias, $\;\delta f^{b}$, comprise the special force, $\;{\hat{f}}^{b}$.

(66) $${\hat{\omega }}_{ib}^{b}=\;\omega_{ib}^{b}+\;\delta \omega_{ib}^{b}=\;\omega_{ib}^{b}+\varepsilon^{b},$$

(67) $${\hat{f}}^{b}=f^{b}+\;\delta f^{b}=f^{b}+\nabla^{b},$$

No matter whether gained from simulation or experiment, the true values of Inertial Measurement Unit (IMU) $\;\omega_{ib}^{b}$ and $\;f^{b}$ are the same. The values of $\;\omega_{ib}^{b}$ and $\;f^{b}$ can be gained from a simulation once the attitude variation and maneuvers of the UUV have been confirmed. The practically-measured data consisted of gyro drifts and the accelerometer bias that was supplied by the IMU in the UUV.

**Figure 10:**
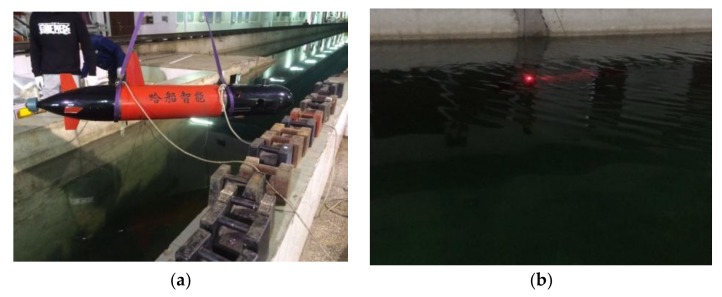
White Dolphin-100 Unmanned Underwater Vehicle: (**a**) White Dolphin-100 UUV ready to be launched into the water; (**b**) White Dolphin-100 UUV during the experiment.

The experiment was conducted in a rectangular pool at our institute in a non-polar region (N45°73′ E127°41′). The follower UUV accomplished a uniform liner motion. The gyro drifts and the accelerometer bias of the follower UUV were extracted from the following UUV that was produced by our laboratory. The follower UUV, called White Dolphin-100 UUV, is shown in Figure 10.

The gyro drifts measured in the non-polar region included gyro constant drifts and gyro random drifts. The gyro constant drifts were 0.03°/h and the gyro random drifts were ${\left(4.094\times 10^{-6}\;\;\mathrm{rad}/s\right)}^{2}\;$, ${\left(4.308\times 10^{-6}\;\;\mathrm{rad}/s\right)}^{2}$ and ${\left(2.386\times 10^{-6}\;\;\mathrm{rad}/s\right)}^{2}$, respectively. The accelerometer biases measured in the non-polar region included accelerometer constant biases and accelerometer random biases. The accelerometer constant biases were $1\times 10^{-6}g_{0}$ and the accelerometer random biases were ${\left(0.00156\;{m/s}^{2}\right)}^{2}$, ${\left(0.001747\;{m/s}^{2}\right)}^{2}$ and ${\left(0.0004063\;{m/s}^{2}\right)}^{2}$, respectively. Other relevant parameters in the experiment were the same as those in the simulation.

The experimental results of the follower UUV, including attitude errors, velocity errors and position errors, are expressed in Figure 10. In this semi-physical simulation, Algorithm 1 was compared with Algorithm 2. Algorithm 1 represents the polar cooperative navigation algorithm of multi-UUVs considering communication delays that has been proposed in this paper. Algorithm 2 is based on conventional AKF which does not consider communication delays.

To describe the experimental results clearly, the RMS errors of attitude errors, velocity errors and position errors are expressed in Table 3.

Comparing the experimental results and the RMS errors, it can be seen that the proposed polar cooperative navigation algorithm of multi-UUVs considering communication delays can be used to estimate states effectively. More accurate information regarding the states can be obtained from the proposed algorithm compared with the conventional algorithm.

### 6.3. Discussions

Conventional SINS is difficult to apply in the polar region. Therefore, the cooperative navigation algorithm based on conventional SINS is not suitable for multi-UUVs in the polar region. Using the grid frame and the UUV characteristics, error equations for the follower UUV that are suitable for the polar environment were established. Using the polar grid algorithm proposed in paper [19], the polar cooperative navigation algorithm was proposed in this paper to solve the navigation problems of multi-UUVs in the polar region.

According to the first comparison simulation (results in Figure 8), communication delay has an impact on results. Because of the communication delay, the estimation errors are divergent, and longer communication delays have larger impacts on the accuracy because the states at time k are corrected by the measurement at time k−i. A bigger D means that the time delay is larger. In addition, the measurement states at time k−i with larger communication delays will have less of a relationship with the states at time k. Low-precision navigation devices have divergent errors and these errors cannot be corrected. Therefore, communication delays cannot be ignored in the polar cooperative navigation algorithm.

**Figure 11:**
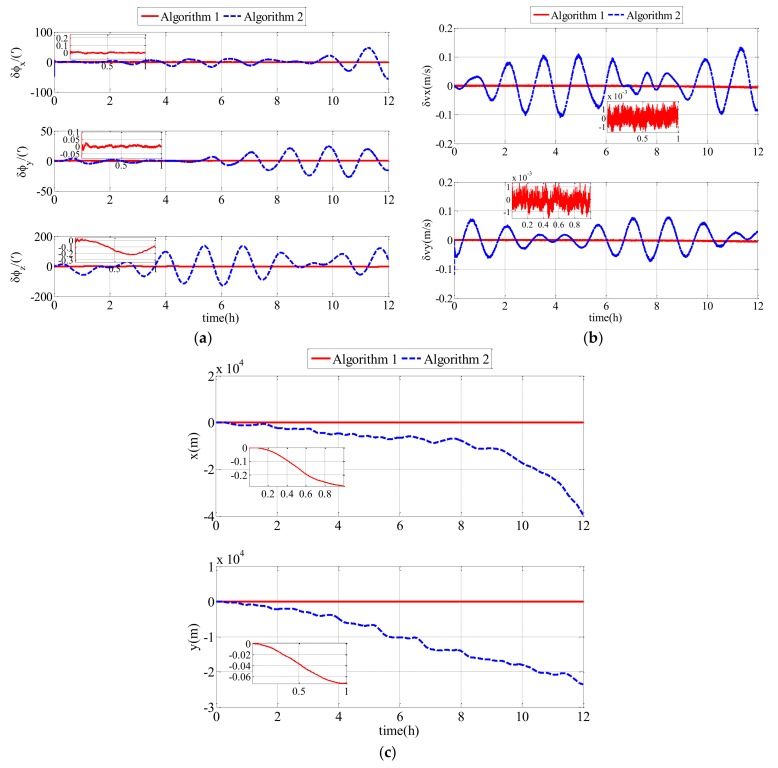
Estimation errors based on Algorithm 1 and Algorithm 2 in the experiment: (**a**) attitude estimation errors; (**b**) velocity estimation errors; (**c**) position estimation errors.

Both the second comparison simulation (results in Figure 9) and the comparison experiment (results in Figure 11) showed that Algorithm 1 is superior to Algorithm 2. Algorithm 1 and Algorithm 2 are suitable for the sailing of multi-UUVs in the polar region. Because of the underwater acoustic communication delay, the information that the follower UUV receives from the leader UUV is not real-time information, rather it is delayed information. In Algorithm 2, the delayed information is misused as real-time information to achieve data fusion. However, in Algorithm 1, the modified AKF considering underwater acoustic communication delay can achieve data fusion using the delayed information. Therefore, there is a bigger drift-away signal or oscillatory result in Algorithm 2 than in Algorithm 1. In Algorithm 1, the communication delay is taken into consideration and the conventional AKF is modified based on the communication delay. Therefore, the communication delay has little impact on the estimation of the errors. The attitude estimation errors, velocity estimation errors and position estimation errors of Algorithm 1 converge quickly and then are stable near zero. Therefore, the polar cooperative navigation algorithm of multi-UUVs considering communication delays is effective and accurate for the sailing of multi-UUVs in the polar region.

A polar cooperative navigation algorithm for multi-UUVs considering communication delays was proposed in this paper. We mainly focused on the construction of the polar cooperative navigation algorithm and only the communication delays were considered in this paper. To simplify the analysis and for the purposes of this paper, some hypothetical ideal situations were included in this paper. For example, the underwater acoustic communication was assumed to be well-performing and reliable. In addition, only communication delays were considered in this paper. There are substantially more complex situations in practical application, including communication interruption, communication lost-packets, communication drop-outs and so on. These situations will influence the accuracy of the cooperative navigation. We believe these problems will be solved with further improvement in underwater acoustic communication and navigation algorithms, which will be our future job.

## 7. Conclusions

A polar cooperative navigation algorithm for multi-UUVs considering communication delays was proposed in this paper. Using UUV characteristics and the environment of the polar region, a polar grid navigation algorithm for a leader UUV and follower UUV was established. The acoustic communication delay was analyzed, and a discretization model for the acoustic communication delay was established. Due to the existence of acoustic communication delays, there are large errors when conventional AKF is used in the polar cooperative navigation of multi-UUVs which consider communication delay. Therefore, the conventional AKF method was modified to consider underwater acoustic communication delays. The simulation and experimental results show that the proposed algorithm can be effectively used for cooperative navigation of multi-UUVs in the polar region.

## Figures and Tables

**Table 1:** RMS errors of polar cooperative navigation of multi-UUVs based on conventional AKF.

Parameters	D1	D2	D3	D4
$\phi_{x}$/(′)	0.9893	0.3130	0.0474	0.0734
$\phi_{y}$/(′)	0.0646	0.1698	0.2496	0.1341
$\phi_{z}$/(′)	3.0777	1.5085	0.8161	0.8719
$v_{x}$/(m/s)	0.0045	0.0083	0.0150	0.0202
$v_{y}$/(m/s)	0.0520	0.0184	0.0401	0.0028
$r_{x}$/(m)	63.667	40.433	21.188	3.120
$r_{y}$/(m)	28.437	21.082	13.080	4.240

**Table 2:** RMS errors of Algorithm 1 and Algorithm 2 in the simulation.

Parameters	Algorithm 1	Algorithm 2
$\phi_{x}$/(′)	0.0229	13.5681
$\phi_{y}$/(′)	0.0291	10.1113
$\phi_{z}$/(′)	1.2218	62.1246
$v_{x}$/(m/s)	0.0021	0.0550
$v_{y}$/(m/s)	0.0022	0.0355
$r_{x}$/(m)	37.3563	1252.2
$r_{y}$/(m)	9.5654	1244.7

**Table 3:** RMS errors of Algorithm 1 and Algorithm 2 in the experiment.

Parameters	Algorithm 1	Algorithm 2
$\phi_{x}$/(′)	0.0242	13.5848
$\phi_{y}$/(′)	0.0266	10.1178
$\phi_{z}$/(′)	1.2140	62.1555
$v_{x}$/(m/s)	0.0021	0.0551
$v_{y}$/(m/s)	0.0022	0.0355
$r_{x}$/(m)	37.1366	1253.2
$r_{y}$/(m)	9.4960	1244.3

## References

[1] Jacobi M. (2015). Autonomous inspection of underwater structures. Robot. Auton. Syst..

[2] Wynn R.B., Huvenne V.A.I., Le Bas T.P., Murton B.J., Connelly D.P., Bett B.J., Ruhl H.A., Morris K.J., Peakall J., Parsons D.R. (2014). Autonomous Underwater Vehicles (AUVs): Their past, present and future contributions to the advancement of marine geoscience. Mar. Geol..

[3] Cao X., Zhu D.Q., Simon X.Y. (2016). Multi-AUV Target Search Based on Bioinspired Neurodynamics Model in 3-D Underwater Environments. IEEE Trans. Neural Netw. Learn. Syst..

[4] Gerigk M.K. (2016). Modeling of Combined Phenomena Affecting an AUV Stealth Vehicle. TransNav Int. J. Mar. Navig. Saf. Sea Transp..

[5] Zhou Q. (2013). All-Earth Inertial Navigation Algorithm for Large Aircraft. Northwest. Polytech. Univ..

[6] Zhou Q., Qin Y.Y., Fu Q.W., Yue Y.Z. (2013). Grid mechanization in Inertial Navigation Syatem for Transpolar Aircraft. J. Northwest. Polytech. Univ..

[7] Cheng J.H., Wang T.D., Guan D.X., Li M.L. (2016). Polar transfer alignment of shipborne SINS with a large misalignment angle. Meas. Sci. Technol..

[8] Xu B., Bai J.L., Hao Y.L., Gao W., Liu Y.L. (2015). The research status and progress of cooperative navigation for multiple AUVs. Acta Autom. Sin..

[9] Liu M.Y. (2014). Cooperative Navigation Technology for Underwater Vehicles.

[10] Matos A., Cruz N. (2005). AUV navigation and guidance in a moving acoustic network. Proc. IEEE/MTS OCEANS Conf. Exhib..

[11] Vaganay J., Leonard J., Curcio J., Willcox J.S. (2004). Experimental validation of the moving long base line navigation concept. Proc. IEEE/OES AUV Conf..

[12] Liu J., Cai B.G., Wang J. (2017). Cooperative localization of connected vehicles: Integrating GNSS with DSRC using a robust cubature Kalman filter. IEEE Trans. Intell. Transp. Syst..

[13] Manzoor S., Lee S., Choi Y. (2017). A Coordinated navigation strategy for multi-robots to capture a target moving with unknown speed. J. Intell. Robot. Syst..

[14] Scheggi S., Aggravi M., Prattichizzo D. (2017). Cooperative navigation for mixed human-robot teams using haptic feedback. IEEE Trans. Hum. Mach. Syst..

[15] Xiao G.D., Wang S.T., Wang B., Deng Z.H. A cooperative navigation method based on USBL. Proceedings of the 2016 China International Conference on Inertial Technology and Navigation.

[16] Xiao G.D., Wang B., Deng Z.H., Fu M., Ling Y. (2017). An Acoustic Communication Time Delays Compensation Approach for Master-Slave AUV Cooperative Navigation. IEEE Sens. J..

[17] Allotta B., Caiti A., Costanzi R., Di Corato F., Fenucci D., Monni N., Pugi L. (2016). Cooperative navigation of AUVs via acoustic communication networking: Field experience with the Typhoon vehicles. Auton. Robots.

[18] Gao W., Yang J., Liu J., Xu B., Shi H.Y. (2014). Cooperative location of multiple UUVs based on hydro-acoustic communication delay. Syst. Eng. Electron..

[19] Yan Z.P., Wang L., Zhang W., Zhou J., Wang M. (2017). Polar Grid Navigation Algorithm for Unmanned Underwater Vehicles. Sensors.

[20] Thong Y.K., Woolfson M.S., Crowe J.A., Hayes-Gill B.R., Challis R.E. (2002). Dependence of inertial measurements of distance on accelerometer noise. Meas. Sci. Technol..

[21] Bian H.W., Jin Z.H., Tian W.F. (2005). Study on GPS attitude determination system aided INS using adaptive Kalman filter. Meas. Sci. Technol..

[22] Huang Y.L., Zhang Y.G. (2017). Robust Student’s t-Based Stochastic Cubature Filter for Nonlinear Systems With Heavy-Tailed Process and Measurement Noises. IEEE Access..

